# Compressed sensing reconstruction for high‐SNR, rapid dissolved ^129^Xe gas exchange MRI

**DOI:** 10.1002/mrm.30312

**Published:** 2024-09-25

**Authors:** Jemima H. Pilgrim‐Morris, Guilhem J. Collier, Ryan S. Munro, Graham Norquay, Neil J. Stewart, Jim M. Wild

**Affiliations:** ^1^ POLARIS, Section of Medical Imaging and Technologies, Division of Clinical Medicine, School of Medicine and Population Health University of Sheffield Sheffield UK; ^2^ Insigneo Institute University of Sheffield Sheffield UK

**Keywords:** compressed sensing, dissolved ^129^Xe, gas exchange, hyperpolarized ^129^Xe, lung MRI, natural abundance ^129^Xe

## Abstract

**Purpose:**

Three‐dimensional hyperpolarized ^129^Xe gas exchange imaging suffers from low SNR and long breath‐holds, which could be improved using compressed sensing (CS). The purpose of this work was to assess whether gas exchange ratio maps are quantitatively preserved in CS‐accelerated dissolved‐phase ^129^Xe imaging and to investigate the feasibility of CS‐dissolved ^129^Xe imaging with reduced‐cost natural abundance (NA) xenon.

**Methods:**

^129^Xe gas exchange imaging was performed at 1.5 T with a multi‐echo spectroscopic imaging sequence. A CS reconstruction with an acceleration factor of 2 was compared retrospectively with conventional gridding reconstruction in a cohort of 16 healthy volunteers, 5 chronic obstructive pulmonary disease patients, and 23 patients who were hospitalized following COVID‐19 infection. Metrics of comparison included normalized mean absolute error, mean gas exchange ratio, and red blood cell (RBC) image SNR. Dissolved ^129^Xe CS imaging with NA xenon was assessed in 4 healthy volunteers.

**Results:**

CS reconstruction enabled acquisition time to be halved, and it reduced background noise. Median RBC SNR increased from 6 (2–18) to 11 (2–100) with CS, and there was strong agreement between CS and gridding mean ratio map values (R^2^ = 0.99). Image fidelity was maintained for gridding RBC SNR > 5, but below this, normalized mean absolute error increased nonlinearly with decreasing SNR. CS increased the mean SNR of NA ^129^Xe images 3‐fold.

**Conclusion:**

CS reconstruction of dissolved ^129^Xe imaging improved image quality with decreased scan time, while preserving key gas exchange metrics. This will benefit patients with breathlessness and/or low gas transfer and shows promise for NA‐dissolved ^129^Xe imaging.

## 1 INTRODUCTION

Pulmonary gas exchange can be assessed with hyperpolarized ^129^Xe MRI via quantification of the signal from ^129^Xe dissolved in the alveolar membrane (M) and capillary red blood cells (RBCs).^1^, ^2^ This provides sensitivity to gas‐transfer limitation in lung diseases such as interstitial lung disease, asthma, and chronic obstructive pulmonary disease (COPD).^1^, ^2^, ^3^, ^4^, ^5^, ^6^, ^7^, ^8^ However, dissolved ^129^Xe MRI is limited by low signal (˜2% that of the gaseous‐phase ^129^Xe signal in the lungs), resulting from the low solubility of xenon in the alveolar membrane and the small volume of the dissolved‐phase compartment when compared with the alveolar airspace.^9^ This is in part overcome by using large dissolved‐phase flip angles, which is possible because of the replenishment of the dissolved‐phase magnetization from the gaseous ^129^Xe reservoir. However, achieving sufficient dissolved‐phase ^129^Xe SNR can still be challenging, especially in lung disease patients who have inherently lower RBC signal due to decreased gas transfer and/or reduced capillary perfusion, who may also struggle to complete the approximately 15‐s breath‐hold.^10^

Compressed sensing (CS) involves using the transform sparsity of images and nonlinear optimization to reconstruct randomly undersampled k‐space data.^11^ The denoising properties of CS can also help improve image SNR and enable reduced scan time for both Cartesian^12^, ^13^, ^14^, ^15^, ^16^ and non‐Cartesian^17^ ^129^Xe imaging. CS reconstruction has recently been demonstrated for radial gas exchange imaging data,^17^ but its effect on the key clinical metrics of gas exchange imaging (i.e., the RBC:M, RBC:Gas, and M:Gas signal ratios) has yet to be studied.

^129^Xe imaging is usually performed with enriched Xe (> 85% ^129^Xe isotopic abundance) due to its increased concentration of the spin ½ isotope of interest when compared with natural abundance (NA, 26% ^129^Xe).^18^ NA Xe is typically an order of magnitude cheaper than enriched,^19^ and together with recent progress in gas polarization,^20^ this provides motivation for NA ^129^Xe imaging. The feasibility of NA ^129^Xe gas‐phase ventilation imaging has been established,^18^ but dissolved ^129^Xe imaging with NA Xe has yet to be demonstrated.

In this work, the gas exchange ratios from both CS and a conventional gridding^21^ reconstruction are compared retrospectively in healthy volunteers, patients with COPD, and patients hospitalized following COVID‐19 infection. We then demonstrate the feasibility of imaging dissolved NA ^129^Xe with CS reconstruction in four healthy volunteers.

## 2 THEORY

Non‐Cartesian k‐space data were reconstructed with CS by solving a nonlinear regularized optimization problem:^11^

(1) $$x={\text{argmin}}_{x}{\left\|\mathrm{Ax}-y\right\|}_{2}+\lambda_{1}{\left\|\Psi x\right\|}_{1}+\lambda_{2}{\left\|\mathrm{Tx}\right\|}_{1}$$

where x is the reconstructed image; y is the acquired k‐space data; $\lambda_{1,2}$ are regularization weights; Ψ is the sparsity operator; and T is the finite difference transform for image total variation. The alternating direction method of multipliers algorithm^22^ was used to solve Eq. (1), where Ψ=I (identity), because hyperpolarized gas lung images have natural sparsity in the image domain due to lack of background signal from surrounding tissue,^12^, ^13^ and A is the nonuniform Fourier transform operator:

(2) A=PF

(3) y=Ax+v

where P is the sampling density; F is the Fourier transform; and v represents measurement noise. In non‐Cartesian gridding reconstruction, x is calculated by convolving the radial k‐space data with a gridding kernel to resample to a Cartesian grid, then applying the inverse fast Fourier transform.^4^, ^21^, ^23^

## 3 METHODS

One dissolved ^129^Xe data set (healthy female, 41 years) was retrospectively analyzed to optimize the CS reconstruction parameters, as in previous work.^12^, ^14^ Images were acquired with a four‐echo 3D radial EPSI sequence^4^ on a 1.5T GE HDx scanner (GE Healthcare, Milwaukee, WI, USA) with a ^129^Xe transmit‐receive vest coil (CMRS, Brookfield, WI, USA). ^129^Xe enriched to 86% Xe isotopic abundance (The Linde Group, UK) was hyperpolarized using an in‐house spin exchange optical pumping system,^20^ and a dose of 0.8–1 L (according to participant height) was inhaled from functional residual capacity. Images were acquired over a 14‐s breath‐hold with a 2‐cm^3^ voxel size and a FOV of 40 cm. A bandwidth of 31.25 kHz was used with a TR of 15 ms, TEs of 0.57/1.27/1.97/2.67 ms, and a flip angle of 22°/0.22° on the dissolved/gas phase. Twenty dummy RF pulses at the start of the sequence were used to eliminate signal from ^129^Xe in the pulmonary veins and acquire calibration spectra.^4^

Image reconstruction and analyses were carried out in *MATLAB* (version 2022a; MathWorks, Natick, MA, USA) on a Windows PC (Intel Core i7‐4790 processor). The gas, RBC, and M resonances were separated in k‐space using matrix inversion and the chemical shifts and T_2_* of the ^129^Xe in RBCs, alveolar membrane, and alveolar airspace obtained from calibration spectra.^4^, ^24^ A Kaiser‐Bessel convolution kernel^21^ (oversampling ratio = 1.2, kernel width = 8) was used to perform conventional gridding reconstruction for each signal, to a 32^3^ reconstructed matrix size. The RBC:M, RBC:Gas, and M:Gas ratio maps were produced after first correcting the gas signal for the differences in flip angle and T_2_* decay during readout between the gas and dissolved xenon. CS reconstruction was performed using the Berkeley Advanced Reconstruction Toolbox (*BART*)^25^ *pics* function to solve Eq. (1) for each ^129^Xe signal. The values of $\lambda_{1}$, $\lambda_{2}$ and the acceleration factor (AF) for random undersampling were chosen empirically for the combination of parameters which minimized the normalized mean absolute error (NMAE) of the dissolved ^129^Xe ratio maps. NMAE was calculated from the masked images, where masking was carried out by applying a noise threshold to the membrane image from gridding reconstruction. The fully sampled data consisted of 934 equidistant radial spokes, which were acquired in a random order. Retrospective CS reconstructions were performed using all data (AF = 1) and with AFs of 2, 3, and 4 by using only the first 467, 311, and 234 radial spokes, respectively.

To evaluate the fidelity of the CS reconstruction, metrics including the NMAE, coefficient of variation (CV), and the pixelwise linear regression of both the ratio maps and the raw images were assessed. SNR was calculated as the ratio of the mean absolute signal within the lung mask to the SD of the real signal from six slices outside of the lung.

The optimized CS and conventional gridding reconstructions were both evaluated retrospectively for a cohort of 41 subjects (Table 1): 13 healthy volunteers, 5 COPD patients, and 23 patients hospitalized post‐COVID‐19 infection (PC), 14 of whom had residual lung abnormalities on CT or structural lung MRI 3 months after hospital admission (PC‐RLA). These participants were imaged using the same parameters as described previously. The mean ratio and CV values from both reconstructions were compared with linear regression and Bland–Altman analysis. Image fidelity was evaluated with NMAE and pixelwise linear regression of the ratio maps.

**TABLE 1:** Subject demographics and the RBC SNR, NMAE, and adjusted R^2^ of the pixelwise linear regression for the CS reconstruction with AF = 2.

		COPD	PC	PC‐RLA	Healthy	Healthy NA
n (F)		5 (3)	9 (2)	14 (2)	16 (5)	4 (2)
Age (years)		60 ± 9	57 ± 9	66 ± 9	40 ± 13	33 ± 8
RBC:M		0.23 ± 0.05	0.21 (0.17, 0.36)	0.19 ± 0.06	0.41 ± 0.08	0.41 ± 0.04
RBC:Gas		0.0019 ± 0.0004	0.0025 ± 0.0006	0.0017 ± 0.0005	0.0040 ± 0.0011	0.0055 ± 0.0010
M:Gas		0.0084 ± 0.0025	0.0115 (0.0088, 0.0176)	0.0097 ± 0.0016	0.0098 ± 0.0024	0.0138 ± 0.0036
RBC SNR	Gridding	2.1 (2.0–7.4)	4.9 (3.6–8.7)	3.4 (1.9–12.6)	9.2 ± 3.3	5.9 ± 1.7
	CS AF = 2	2.7 (2.3–18.9)	9.3 ± 2.4	5.3 (2.1–28.4)	27.0 (10.2–100.0)	19.8 ± 10.3
NMAE (%)	RBC:M	27.6 ± 9.6	17.2 ± 3.9	19.5 ± 9.8	9.9 ± 3.3	15.0 ± 4.2
	RBC:Gas	33.6 (12.7–33.9)	18.8 ± 4.0	21.5 ± 9.5	10.5 ± 3.2	17.4 ± 7.7
	M:Gas	8.9 ± 1.8	5.8 ± 1.0	6.3 ± 2.0	5.6 ± 1.3	10.3 ± 7.5
R^2^	RBC:M	0.40 (0.30–0.81)	0.61 ± 0.08	0.68 ± 0.18	0.71 ± 0.11	0.54 ± 0.02
	RBC:Gas	0.54 ± 0.18	0.62 ± 0.06	0.61 ± 0.15	0.78 (0.56–0.88)	0.62 (0.19–0.68)
	M:Gas	0.75 ± 0.02	0.83 ± 0.07	0.72 ± 0.17	0.83 (0.40–0.94)	0.83 (0.29–0.88)

*Note*: Normally distributed variables (as determined with Shapiro‐Wilks tests) are given as mean ± SD, whereas nonnormally distributed variables are given as median (minimum, maximum). Gas‐exchange ratio values are from gridding reconstruction. “Healthy NA” refers to the healthy subjects who underwent NA‐dissolved ^129^Xe imaging; 3 of these subjects were in common with the healthy group.

Abbreviations: AF, acceleration factor; CS, compressed sensing; COPD, chronic obstructive pulmonary disease; NA, natural abundance ^129^Xe; NMAE, normalized mean absolute error; PC, post‐COVID‐19; PC‐RLA, post‐COVID‐19 residual lung abnormalities; R^2^, coefficient of determination; RBC:Gas, red blood cell to gas signal ratio; RBC:M, red blood cell to membrane signal ratio.

Correction added after online publication 25th October 2024. Due to a publisher's error, Table 1 has been correctly formatted in this version.

Four healthy volunteers were then prospectively imaged (with the fully sampled sequence) with 0.8–1‐L hyperpolarized NA ^129^Xe (dose equivalent volume^19^ of ˜80 mL for the 1‐L dose, with 30% polarization) on a 1.5T GE Artist scanner to assess the feasibility of NA‐dissolved ^129^Xe CS imaging. 3 of 4 participants additionally underwent dissolved imaging with the same volume of enriched ^129^Xe (dose equivalent volume ˜260 mL) in the same scanning session, and these data were added to the healthy volunteer cohort.

All imaging protocols were performed under the approval of the UK National Research Ethics Committee (REC reference numbers 17/LO/0725, 9/LO/1115 and 22/NW/0009) and University of Sheffield ethics UOS030529.

## 4 RESULTS

### 4.1 CS optimization

**FIGURE 1:**
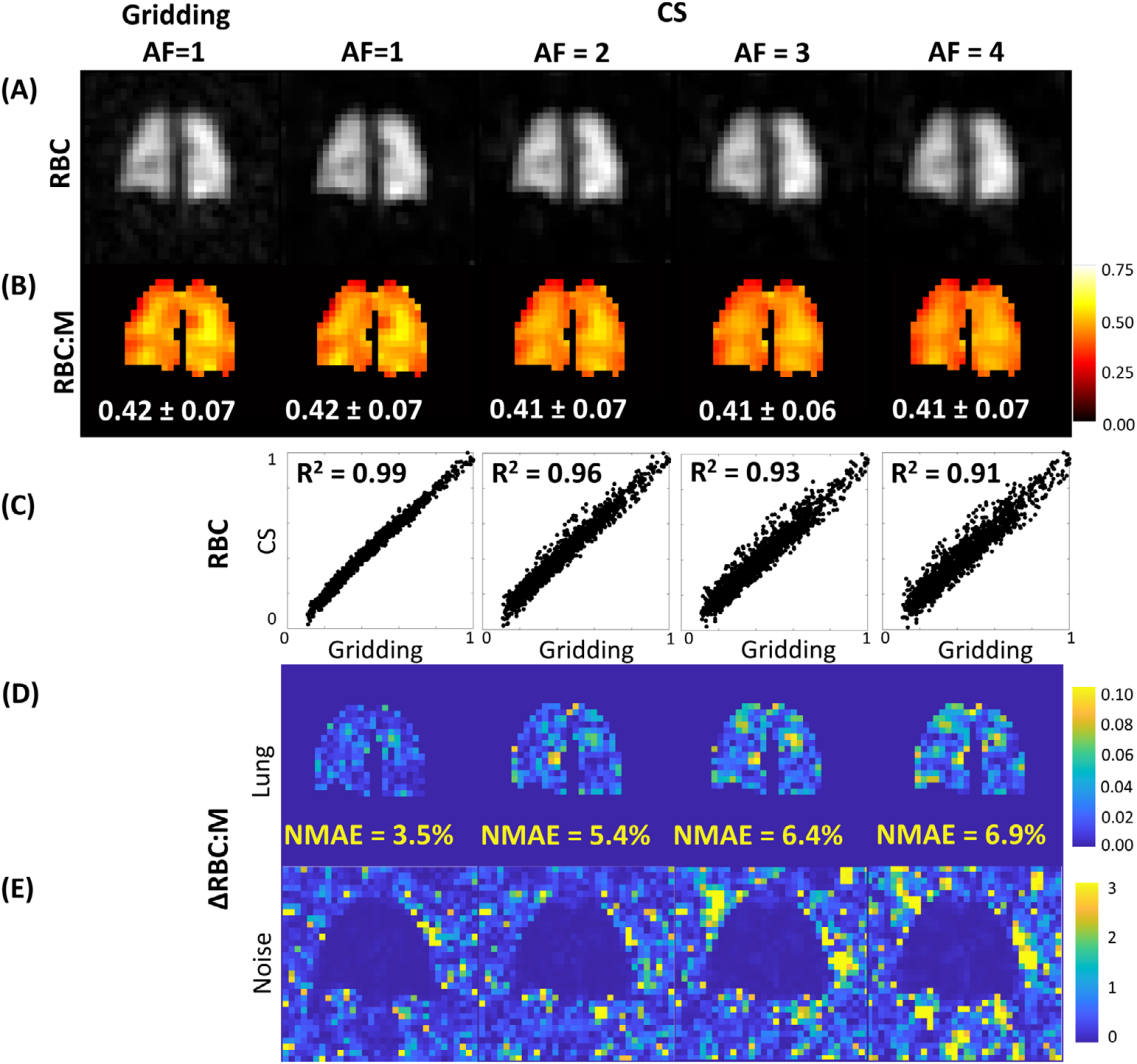
(A) Red blood cell (RBC) signal. (B) RBC to alveolar membrane signal ratio (RBC:M) from conventional gridding reconstruction (100% sampling) and compressed‐sensing (CS) reconstruction with acceleration factor (AF) = 1–4. (C) The corresponding linear regression of the normalized pixelwise RBC signal. The absolute difference between the RBC:M maps from gridding and CS reconstruction are shown for each AF, for inside (D) and outside (E) the lung mask. R^2^, adjusted coefficient of determination.

To balance image fidelity and noise suppression, $\lambda_{1}$ = 0.003 and $\lambda_{2}$ = 0.0003 were chosen for the optimized CS implementation. These parameters provided good agreement between the gridding and CS‐reconstructed mean ratio values at each AF (Figure 1, Table S1) and worked well over a range of other lung images tested. As the AF increased from 1 to 4, NMAE increased (RBC:M, 3.5%–6.9%; RBC:Gas, 5.2%–8.3%; M:Gas, 4.2%–4.8%) and CV decreased (RBC:M, 0.17–0.16; RBC:Gas, 0.29–0.22; M:Gas, 0.28–0.20), as expected from the effect of CS smoothing and loss of information. Linear regression of the normalized pixelwise signal showed high R^2^ values, which decreased with increasing AF (RBC, 0.99–0.91; M, 0.98–0.90; Gas, 0.98–0.87). As a compromise between image fidelity and scan time reduction, AF = 2 was chosen for the optimized CS implementation. With these optimized parameters, the distribution of the gas exchange ratios was preserved, resulting in similar maps and histograms (Figure S1). The CS reconstruction time was similar to that of gridding (< 5 min).

### 4.2 Retrospective analysis

**FIGURE 2:**
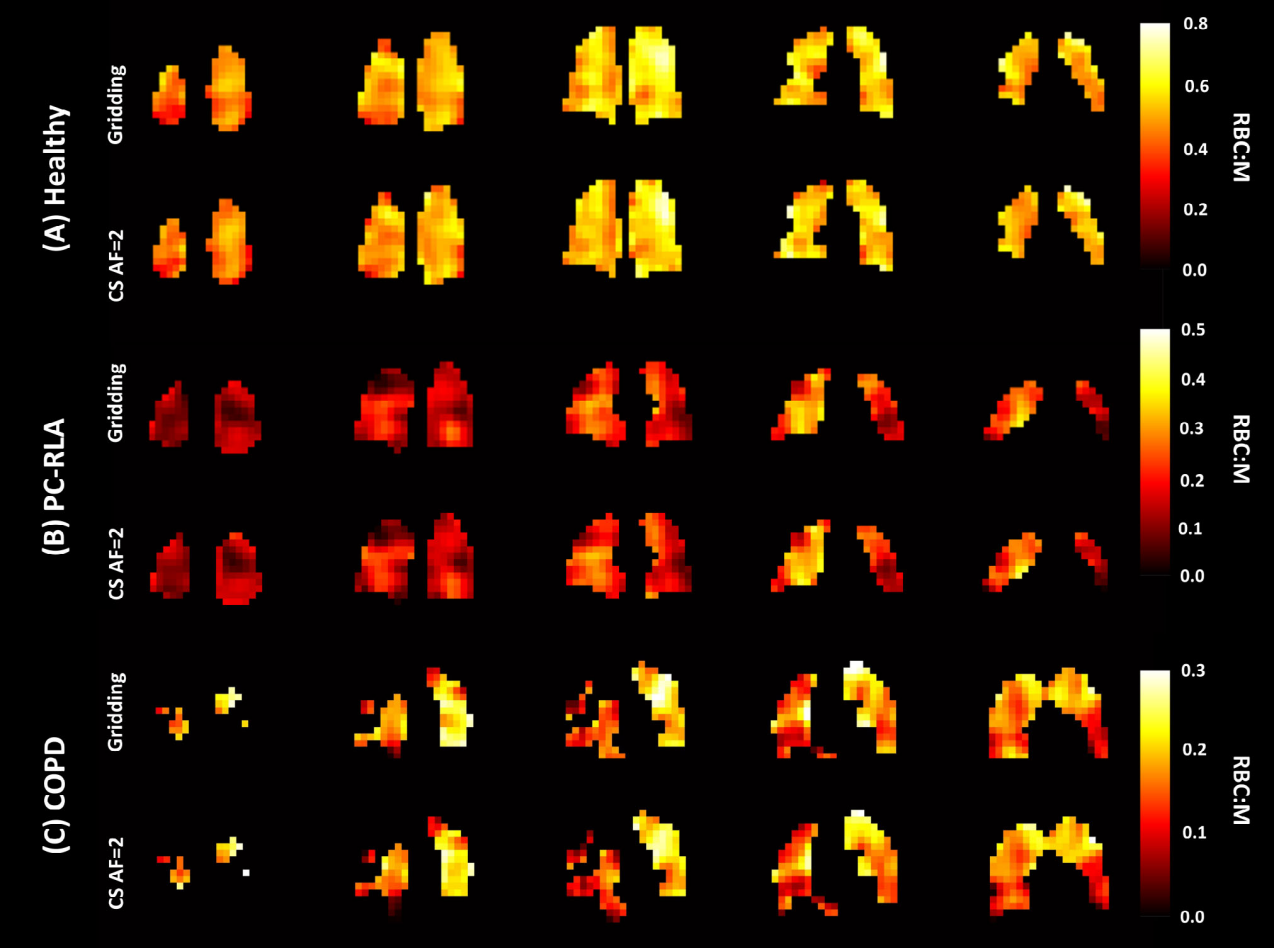
Red blood cell to alveolar membrane ratio (RBC:M) maps from gridding and compressed‐sensing (CS) reconstruction for five lung slices (posterior to anterior) for a healthy participant (A), a post‐COVID‐19 patient with residual lung abnormalities (PC‐RLA) (B), and a patient with chronic obstructive pulmonary disease (COPD) (C). For the 2 lung disease patients, regions of RBC transfer defect are preserved in the CS maps. AF, acceleration factor.

**FIGURE 3:**
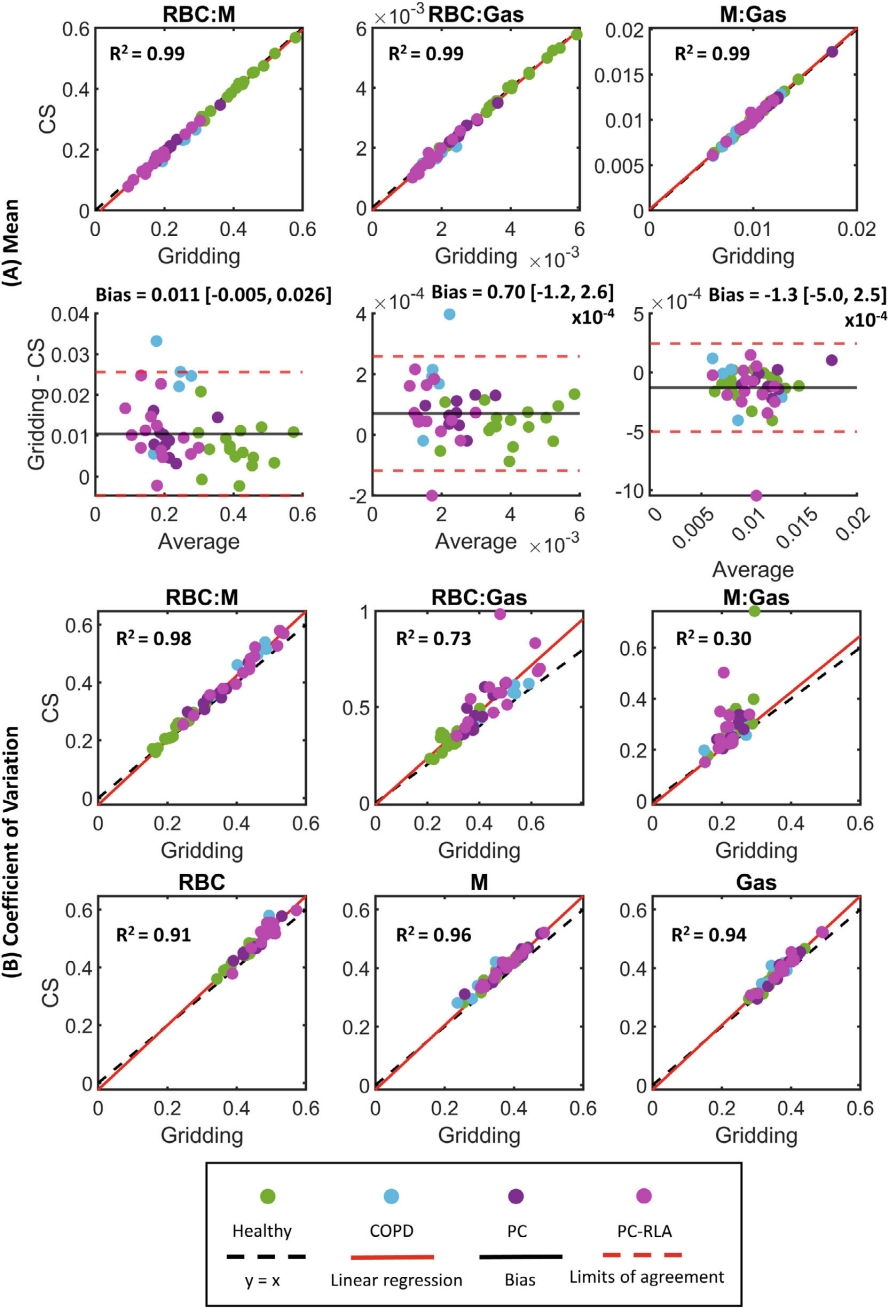
(A) Linear regression and Bland–Altman plots of whole‐lung mean for red blood cell to membrane signal ratio (RBC:M), RBC to gas signal ratio (RBC:Gas), and membrane to gas signal ratio (M:Gas). (B) Linear regression plots of coefficient of variation (CV) for RBC:M, RBC:Gas, M:Gas, and the RBC, M, and Gas signals. These were calculated from gridding and compressed‐sensing (CS) reconstructed ^129^Xe signal maps for the healthy volunteer and patient cohort. PC, post‐COVID‐19; PC‐RLA, post‐COVID‐19 patient with residual lung abnormalities.

Patient demographics and key results are given in Table 1, and example ratio maps are shown in Figure 2. The mean gas exchange ratio values were preserved with the CS reconstruction, even for patients with very low RBC image SNR (Figure 3A). The R^2^ values for each ratio were high (0.99), and Bland–Altman analysis showed evenly spread residuals. The NMAE values between the gridding and CS‐derived mean ratios were 3.8% for RBC:M, 3.3% for RBC:Gas, and 1.5% for M:Gas. The CV values from gridding and CS reconstruction were similar for RBC:M, RBC, M and Gas, with R^2^ > 0.91 and small Bland–Altman biases (Figure 3B). For RBC:Gas and M:Gas, CS CV was significantly higher than that of gridding (Wilcoxon signed rank exact test: *p* < 1 × 10^−8^). A small number of outliers (driven by low signal pixels at the periphery of the lungs with very high ratio values) resulted in lower R^2^ values of 0.73 and 0.30, respectively.

The pixelwise linear regression between the gridding and CS derived ratio maps yielded R^2^ = 0.65 ± 0.16 for RBC:M, 0.65 ± 0.14 for RBC:Gas, and 0.78 (0.40–0.94) for M:Gas, across the 44 subjects. The NMAE values were higher in this cohort than from the CS simulations, and a median NMAE of 15.0% was found for RBC:M. The NMAE values for RBC:M, RBC:Gas, and M:Gas were observed to scale nonlinearly with the dissolved ^129^Xe gridding SNR values (Figure S2A–D). In particular, when gridding RBC SNR dropped below five, NMAE increased sharply; overall the relationship was found to be best explained by a power law:

(4) $$\text{NMAE}=a{\mathrm{SNR}}^{k}$$

where a is a scaling constant, and k < 0 is the exponent of the power law.

CS suppressed noise such that RBC SNR increased by an average factor of 2 for the retrospective analysis cohort. A 3‐fold SNR increase was found for the healthy volunteers' CS images. The relationship between CS and gridding RBC SNR was nonlinear and followed a power law with k = 1.82 (Figure S2E).

### 4.3 Natural‐abundance ^129^Xe

**FIGURE 4:**
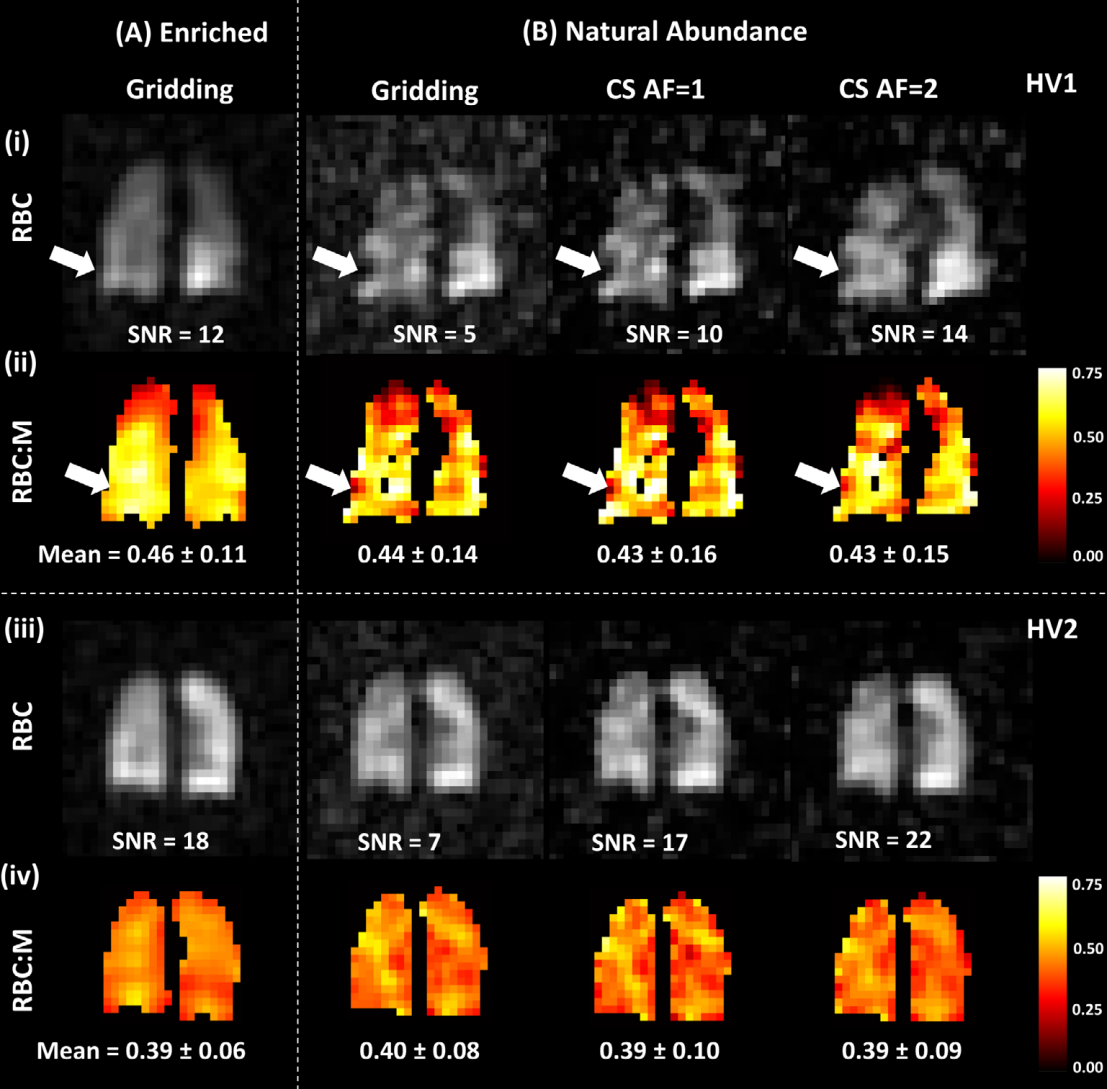
Enriched (A) and natural abundance (NA) (B) dissolved ^129^Xe red blood cell (RBC) signal (i, iii) and RBC to membrane signal ratio (RBC:M) (ii, iv) maps for a central lung slice in 2 healthy volunteers (HV). The mean ± SD of the RBC:M ratio maps are given below. The NA signal images and ratio maps exhibited greater heterogeneity than those from enriched ^129^Xe imaging. For HV1, there are areas of very low/no signal, such as in the lower‐right lung (*white arrow*), which appear as RBC transfer defects in the RBC:M maps and are not present in the enriched ^129^Xe image.

As expected from the lower isotopic concentration, the SNR of the NA‐dissolved ^129^Xe images was worse than those acquired with enriched ^129^Xe (Figure 4). For the 3 participants who had enriched and NA dissolved ^129^Xe imaging in the same session, the ratio of enriched to NA gridding RBC image signal was 3.0 (compared with a 3.3 difference in ^129^Xe concentration). CS was successful at suppressing noise in the NA ^129^Xe images; at AF = 2, RBC SNR was increased 3‐fold compared with gridding reconstruction (Table 1). The mean ratio values were similar between the gridding (RBC:M = 0.41 ± 0.04, RBC:Gas = 0.0055 ± 0.0010, M:Gas = 0.014 ± 0.004) and CS AF = 2 reconstructed maps (RBC:M = 0.41 ± 0.04, RBC:Gas = 0.0056 ± 0.0010, M:Gas = 0.014 ± 0.004), and the R^2^ and NMAE values were comparable to those from enriched ^129^Xe imaging, although the M:Gas NMAE was higher.

The top panel of Figure 4 shows the enriched and NA RBC images for a healthy male participant. The NA images exhibited more heterogeneity than the enriched image, with CV = 0.32 (gridding), 0.37 (CS AF = 1), and 0.35 (CS AF = 2), compared with CV = 0.23 from enriched ^129^Xe imaging (gridding). In a second healthy volunteer (Figure 4, bottom panel), the NA RBC gridding image SNR (6.9) was higher than that of the first healthy volunteer (4.8), and there was a closer resemblance between the enriched and NA xenon spectroscopic images.

## 5 DISCUSSION

We have presented a CS reconstruction method for dissolved ^129^Xe spectroscopic imaging data using the open‐source *BART* software.^25^ We chose to optimize the CS parameters based on the gas exchange ratio maps of one healthy data set, in addition to the RBC, M, and gas signals, because it is the ratios that are ultimately used in current approaches of clinical interpretation. Although the optimal parameters may be slightly subject‐dependent, in our experience the changes are small. We empirically selected AF = 2 for our optimized CS reconstruction, because this provided the best balance between image fidelity and scan time reduction. With this AF, only 467 radial spokes are required; therefore, in a prospectively undersampled implementation, the breath‐hold could be reduced to 7 s compared with the 14 s used in our center routinely.^4^ A shorter scan time should be more comfortable for dyspneic and pediatric patients.

SNR increased with increasing AF for both the optimization and NA reconstructions, despite the greater level of undersampling. This is likely because, with a higher AF, more of the radial spokes from the end of the acquisition that have undergone increased RF pulse–induced depolarization^26^ and oxygen‐related T_1_ decay are discarded. Sampling the spokes randomly over the acquisition reversed this trend (Figure S3).

Both the gridding and CS images were masked based on the gridding M image; however, this occasionally led to outliers in the ratio maps. Some low signal voxels at the periphery of the gridding masks may have been suppressed as noise in the CS reconstruction but were still included in the ratio maps. In a prospective CS implementation, masking would be performed using the CS M images instead, alleviating this issue.

CS reconstruction improved SNR by suppressing image noise. Our results agree with those of Plummer et al.,^17^ who found an increase in the overall (RBC + M) dissolved‐phase ^129^Xe SNR with CS, and good preservation of median dissolved‐phase to gas signal ratio in healthy volunteers and patients who had undergone a hematopoietic stem cell transplant. Here, we have gone further by separating the dissolved‐phase ^129^Xe signal into the discrete RBC and M contributions, and examining the effect of the CS reconstruction on the gas exchange ratios in healthy participants and patients with lung disease. Although the increase in RBC SNR was less for the lung disease patients than the 3‐fold increase seen for the healthy volunteers, the healthy participants had reasonably high gridding SNR, whereas this was low (< 5) for many of the patients. The CS SNR enhancement was dependent on the input (gridding) SNR. When this is higher, there is increased separation between the signal in the lung region and noise in the image domain representation, providing a better ground truth for the iterative CS reconstruction and leading to better denoising performance. Although outside the scope of this work, a comparison of the denoising effect of CS with novel denoising techniques such as tensor Marchenko‐Pastur principle component analysis,^27^ global local higher‐order singular value decomposition^28^ and deep learning^29^, ^30^ would be an interesting future avenue of research.

For patients with low dissolved‐phase ^129^Xe signal, although CS reconstruction provided a beneficial increase in SNR, there was an associated increase in NMAE between the CS and gridding ratio maps; for RBC gridding image SNR < 5, errors for RBC:M rose above 15%. Nevertheless, in cases of such low SNR, it is not appropriate to use the gridding images as the ground truth, because comparison of signal with noisy images is not reliable. Given the lack of an alternative ground truth, and therefore the uncertainty in the CS algorithm performance when signal is low, we cannot reliably conclude that the CS reconstruction is capable of recovering RBC data with raw SNR < 5. This highlights the limitations of retrospective denoising and the importance of techniques to prospectively increase dissolved‐phase ^129^Xe SNR, such as increasing the polarization and dose/^129^Xe fraction, and using high‐sensitivity RF coils where feasible.

Even for higher SNR cases, NMAE values were reasonably high, sometimes exceeding 10%. However, it is important to consider the NMAE values in the context of the repeatability of the gas exchange ratio measurements themselves. Intersession CV values for the mean ratios have been reported to be between 11% and 20% by Hahn et al.^31^ in 13 healthy volunteers scanned at two time points, 1 month apart, with the 1‐point Dixon method.^1^, ^32^ In a study of 18 healthy participants and 14 with COPD, Garrison et al. found intrasession CV values of 7%–13%.^33^ Therefore, here, for SNR > 5, the NMAE values of the ratio maps are similar to their repeatability metrics.

NA‐dissolved ^129^Xe imaging had not previously been explored due to the 3‐fold lower signal strength of NA when compared with enriched ^129^Xe. Here, we have demonstrated the feasibility of dissolved‐NA ^129^Xe imaging for the first time in healthy subjects. We used advancements in polarization technology and the denoising effect of CS to achieve an acceptable RBC image SNR from the same volume of NA ^129^Xe as was used for enriched dissolved ^129^Xe imaging. A limitation of this work is that NA imaging was only acquired in a small number of healthy participants who did not have any gas exchange limitation. Further prospective evaluation in increased subject numbers, including patients with lung disease, is required to fully evaluate the differences between enriched and NA‐dissolved ^129^Xe imaging and to assess the feasibility of this approach when gas transfer and/or RBC SNR is low. These initial results are promising for clinical implementation of low‐cost NA‐dissolved ^129^Xe imaging.

## 6 CONCLUSIONS

CS reconstruction of dissolved ^129^Xe spectroscopic imaging improved image SNR and enabled reduced scan time, while preserving mean ratio values. This benefits patients with breathlessness and/or low gas transfer, allowing for faster gas exchange imaging. Therefore, CS reconstruction may be suitable as a replacement for conventional gridding in our dissolved ^129^Xe image reconstruction pipeline, although caution should be taken with patient groups with very low RBC SNR, such as COPD and interstitial lung disease. Preliminary results with NA‐dissolved ^129^Xe imaging in healthy participants suggest that reduced cost NA gas exchange imaging could be made feasible using CS.

## Supporting information


**Figure S1.** (A) Ratio maps from five slices of the lung for the healthy data set used to optimize the compressed‐sensing (CS) parameters. (B) Histograms of the ratio maps from gridding and CS reconstruction (acceleration factor (AF) = 2) for red blood cell to membrane signal ratio (RBC:M), red blood cell to gas signal ratio (RBC:Gas), and membrane to gas signal ratio (M:Gas).
**Figure S2.** A nonlinear power law relationship was found between the normalized mean absolute error (NMAE) of the compressed‐sensing (CS) ratio maps, relative to the gridding ratio maps, and the dissolved ^129^Xe gridding SNR (A–D) and the SNR of the red blood cell (RBC) image derived from gridding and CS (E). The fitted power law parameters, a and k, are given along with their 95% confidence intervals. RMSE, RMS error.
**Figure S3.** SNR of the signal from ^129^Xe dissolved in the red blood cells (RBCs) and the gaseous‐phase ^129^Xe at acceleration factor (AF) = 1, 2, 3, and 4 for a healthy participant (A,B) and a patient with chronic obstructive pulmonary disease (COPD) (C,D). Two different sampling patterns were compared for both compressed sensing (CS) and gridding: consecutive temporal ordering of the chosen spokes (taking the first half/third/quarter) and random temporal spoke ordering (taking spokes from throughout the acquisition to “average out” the polarization decay). Note the logarithmic y‐axis scale.
**Table S1.** Compressed‐sensing (CS) reconstruction parameters for the healthy volunteer data set used in the optimization process for acceleration factor (AF) = 1, 2, 3, and 4. The mean ratio values and coefficient of variation (CV) from conventional gridding reconstruction are included for comparison.

## Data Availability

The open‐source code and an example data set to perform dissolved ^129^Xe compressed sensing reconstruction can be found at https://github.com/POLARIS‐Sheffield/xe_cs_recon.
