# Supplementary material for: Compressed sensing reconstruction for high‐SNR, rapid dissolved 129Xe gas exchange MRI
Source: Magn Reson Med. 2024 Sep 25;93(2):741–50. doi: 10.1002/mrm.30312 (PMC11604844; doi:10.1002/mrm.30312)
Supplement: Supplementary file 1 — Figure S1. (A) Ratio maps from five slices of the lung for the healthy data set used to optimize the compressed‐sensing (CS) parameters. (B) Histograms of the ratio maps from gridding and CS reconstruction (acceleration factor (AF) = 2) for red blood cell to membrane signal ratio (RBC:M), red blood cell to gas signal ratio (RBC:Gas), and membrane to gas signal ratio (M:Gas). Figure S2. A nonlinear power law relationship was found between the normalized mean absolute error (NMAE) of the compressed‐sensing (CS) ratio maps, relative to the gridding ratio maps, and the dissolved 129Xe gridding SNR (A–D) and the SNR of the red blood cell (RBC) image derived from gridding and CS (E). The fitted power law parameters, a and k, are given along with their 95% confidence intervals. RMSE, RMS error. Figure S3. SNR of the signal from 129Xe dissolved in the red blood cells (RBCs) and the gaseous‐phase 129Xe at acceleration factor (AF) = 1, 2, 3, and 4 for a healthy participant (A,B) and a patient with chronic obstructive pulmonary disease (COPD) (C,D). Two different sampling patterns were compared for both compressed sensing (CS) and gridding: consecutive temporal ordering of the chosen spokes (taking the first half/third/quarter) and random temporal spoke ordering (taking spokes from throughout the acquisition to “average out” the polarization decay). Note the logarithmic y‐axis scale. Table S1. Compressed‐sensing (CS) reconstruction parameters for the healthy volunteer data set used in the optimization process for acceleration factor (AF) = 1, 2, 3, and 4. The mean ratio values and coefficient of variation (CV) from conventional gridding reconstruction are included for comparison. [file MRM-93-741-s001.docx]

Supporting Information

Table S1: CS reconstruction parameters for the healthy volunteer dataset used in the optimization process for AF = 1, 2, 3 and 4. The mean ratio values and CV from conventional gridding reconstruction are included for comparison.

|  | | RBC:M | RBC:Gas | M:Gas |
| --- | --- | --- | --- | --- |
| Mean | Gridding | 0.42 ± 0.07 | 0.0040 ± 0.0010 | 0.0098 ± 0.0021 |
|  | CS AF=1 | 0.42 ± 0.07 | 0.0041 ± 0.0012 | 0.0100 ± 0.0028 |
|  | CS AF=2 | 0.41 ± 0.07 | 0.0041 ± 0.0011 | 0.0099 ± 0.0025 |
|  | CS AF=3 | 0.41 ± 0.06 | 0.0041 ± 0.0010 | 0.0099 ± 0.0022 |
|  | CS AF=4 | 0.41 ± 0.07 | 0.0041 ± 0.0009 | 0.0099 ± 0.0020 |
| CV | Gridding | 0.16 | 0.24 | 0.22 |
|  | CS AF=1 | 0.17 | 0.29 | 0.28 |
|  | CS AF=2 | 0.17 | 0.26 | 0.25 |
|  | CS AF=3 | 0.16 | 0.23 | 0.22 |
|  | CS AF=4 | 0.16 | 0.22 | 0.20 |
| NMAE (%) | CS AF=1 | 3.5 | 5.2 | 4.2 |
|  | CS AF=2 | 5.4 | 6.2 | 4.2 |
|  | CS AF=3 | 6.4 | 7.3 | 4.4 |
|  | CS AF=4 | 6.9 | 8.3 | 4.8 |
| R^2^ | CS AF=1 | 0.92 | 0.87 | 0.88 |
|  | CS AF=2 | 0.81 | 0.78 | 0.82 |
|  | CS AF=3 | 0.73 | 0.83 | 0.89 |
|  | CS AF=4 | 0.69 | 0.79 | 0.90 |
|  | | RBC | M | Gas |
| R^2^ | CS AF=1 | 0.99 | 0.98 | 0.98 |
|  | CS AF=2 | 0.96 | 0.95 | 0.95 |
|  | CS AF=3 | 0.93 | 0.92 | 0.91 |
|  | CS AF=4 | 0.91 | 0.90 | 0.87 |

*Abbreviations: CS = compressed sensing, AF = acceleration factor, CV = coefficient of variation, NMAE = mean absolute error, RBC:M = red blood cell to membrane signal ratio, RBC:Gas = red blood cell to gas signal ratio, M:Gas = membrane to gas signal ratio, R^2^ = coefficient of determination.*


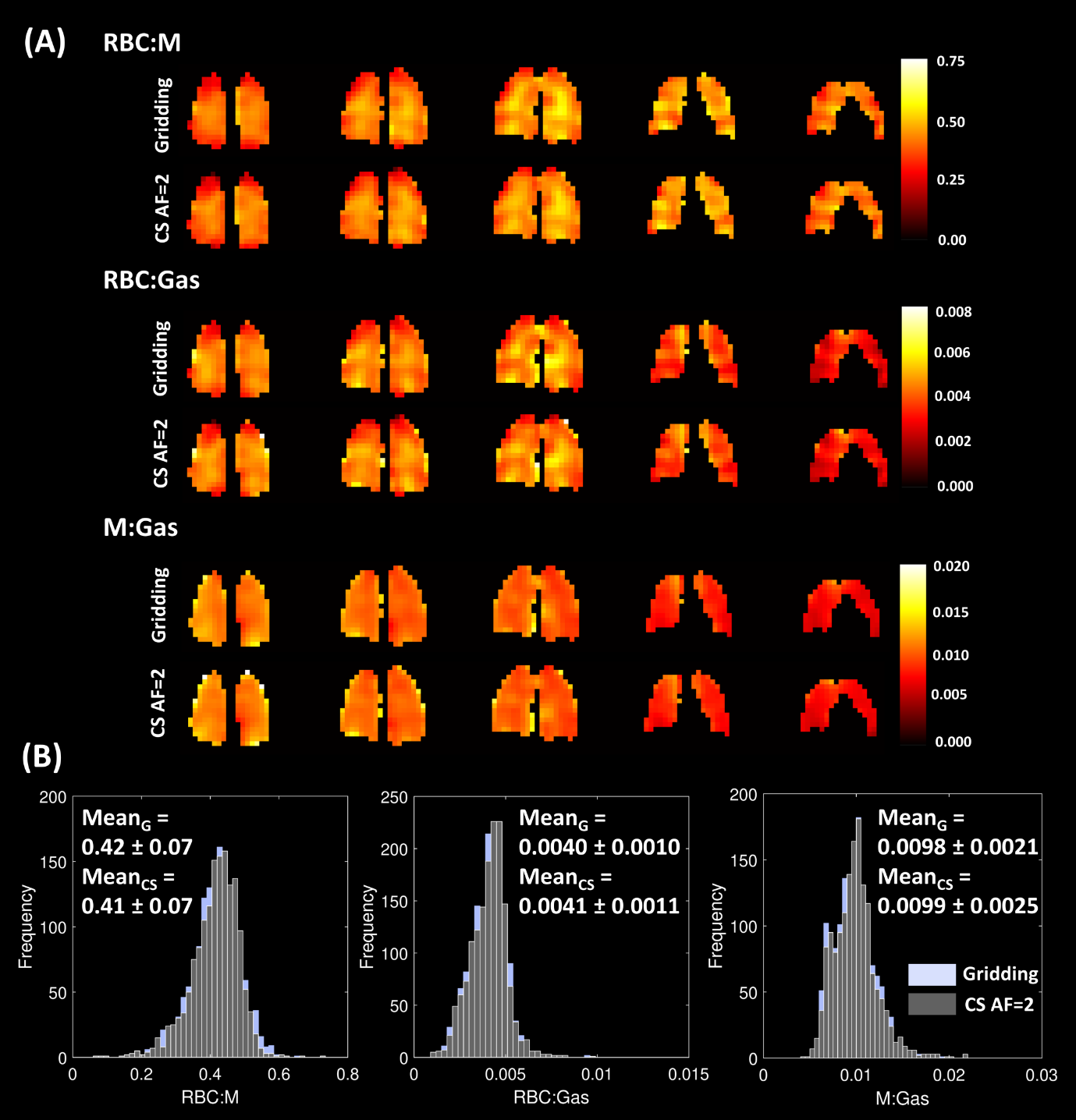


Figure S1: (A) Ratio maps from five slices of the lung for the healthy dataset used to optimize the CS parameters and (B) histograms of the ratio maps from gridding and CS reconstruction (AF=2) for RBC:M, RBC:Gas and M:Gas.


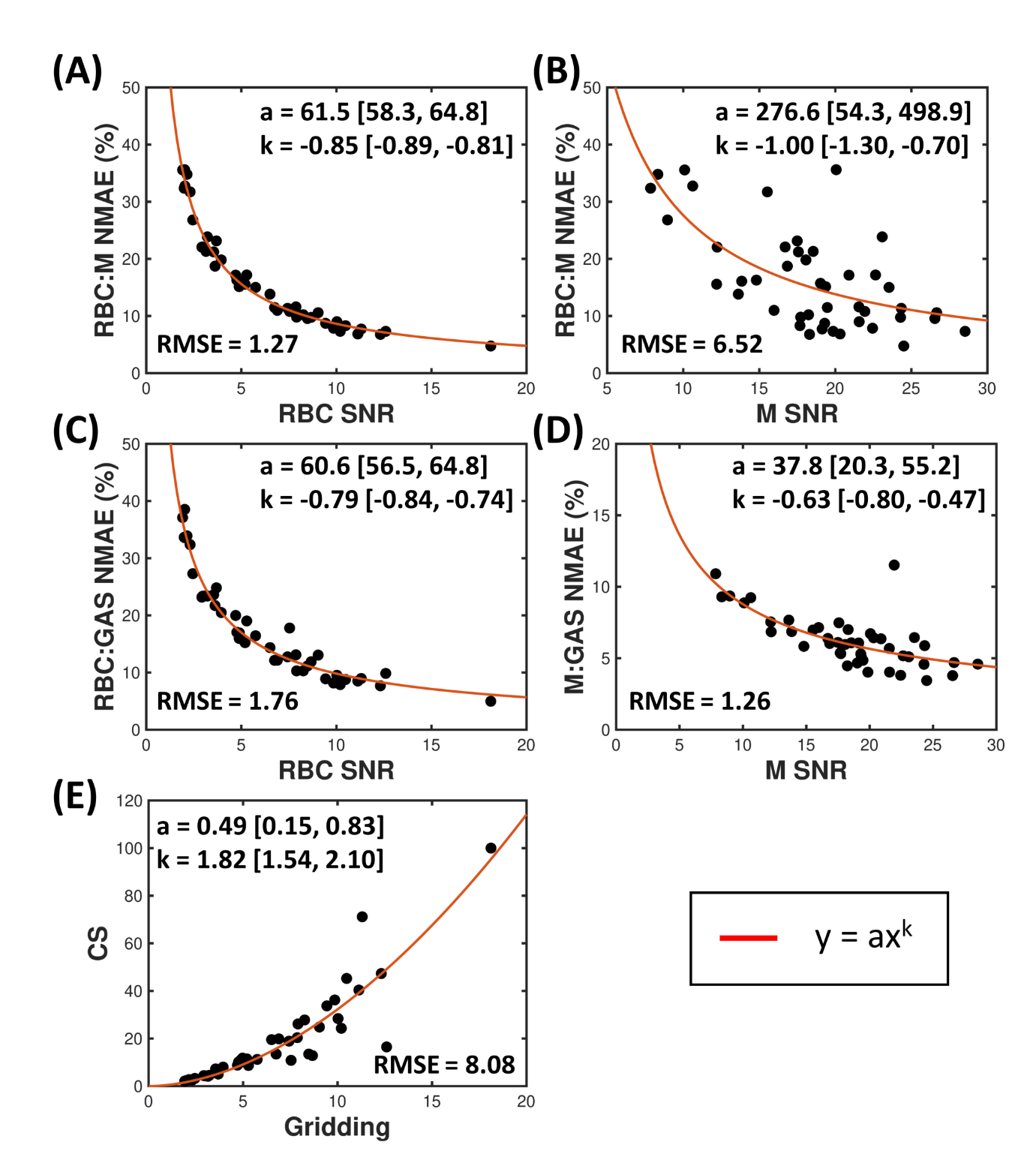


Figure S2: A nonlinear power law relationship was found between the NMAE of the CS ratio maps, relative to the gridding ratio maps, and the dissolved ^129^Xe gridding SNR (A-D) and the SNR of the RBC image derived from gridding and CS (E). The fitted power law parameters, $a$ and $k$, are given along with their 95% confidence intervals. RMSE = root mean squared error.


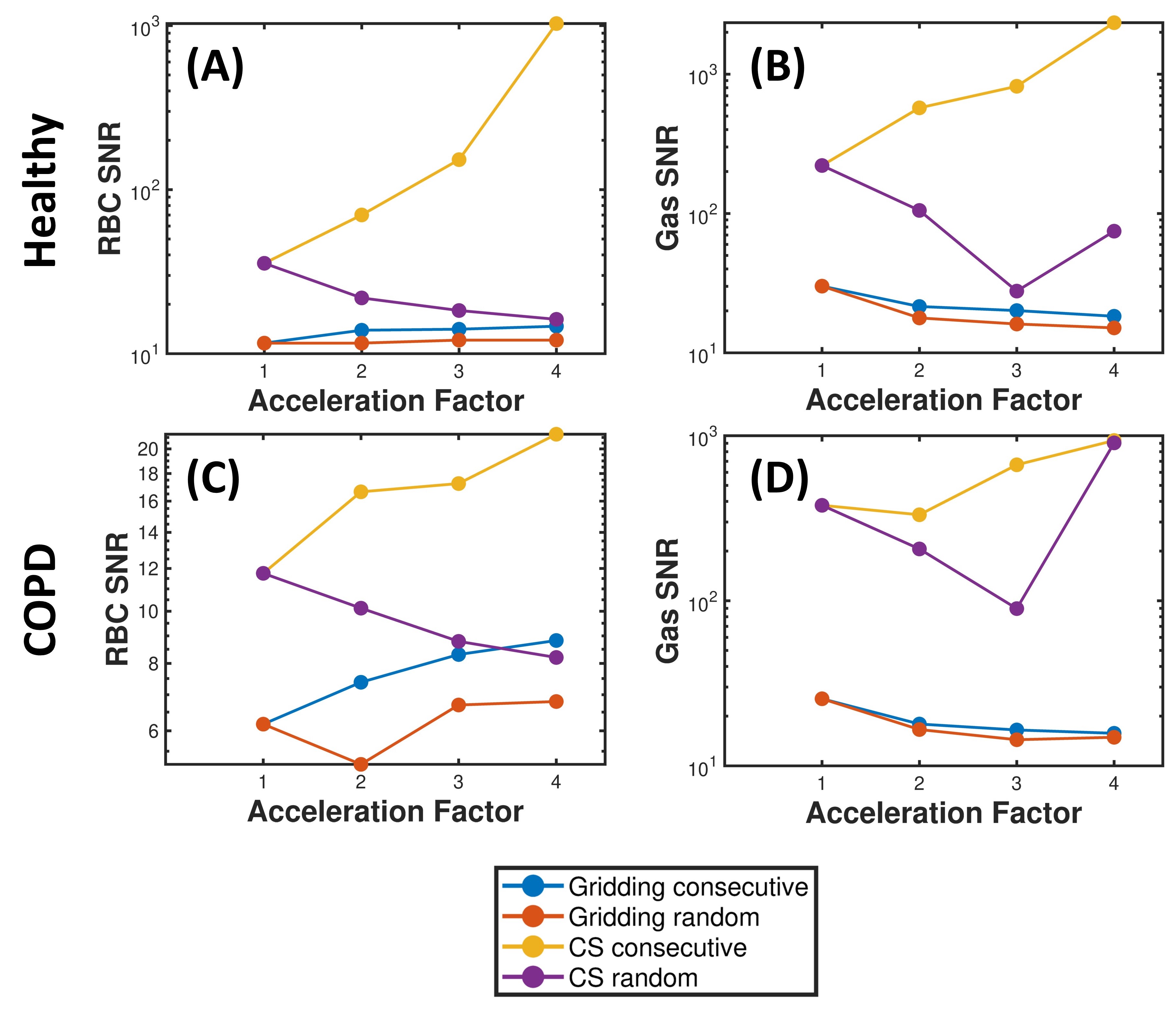


Figure S3: SNR of the signal from ^129^Xe dissolved in the RBCs and the gaseous-phase ^129^Xe at AF = 1, 2, 3 and 4 for (A-B) a healthy participant and (C-D) a patient with COPD. Two different sampling patterns were compared for both CS and gridding: consecutive temporal ordering of the chosen spokes (taking the first half/third/quarter) and random temporal spoke ordering (taking spokes from throughout the acquisition to “average out” the polarization decay). Note the logarithmic *y*-axis scale.

Using consecutive spoke ordering increases the SNR relative to that found using random temporal ordering. This is because, with consecutive ordering, the latter 1/2, 2/3 or 3/4 of the radial spokes which have undergone more T_1_ and RF pulse induced depolarization, are discarded. As more spokes are discarded, the average signal of the spokes selected for reconstruction increases and this partly explains why SNR is seen to increase with AF. This effect is also due to the nonlinear noise suppression of the compressed sensing algorithm.^1^ However, the high SNR values at AF = 4 are not ‘true’ SNR as per its standard definition, and may be due to over-smoothing and/or reconstruction errors from heavy undersampling and from the conventional standard deviation-based approach to calculate the SNR. This may not be appropriate for highly accelerated CS images where the noise appears as a “flat” background.

References

1. Lustig M, Donoho D, and Pauly JM, *Sparse MRI: The application of compressed sensing for rapid MR imaging.* Magnetic Resonance in Medicine, 2007. **58**(6): p. 1182-95.
